# Preoperative fibrinogen-to-lymphocyte ratio as a prognostic biomarker for non-muscle-invasive bladder cancer

**DOI:** 10.3389/fonc.2026.1707696

**Published:** 2026-01-22

**Authors:** Xueqiao Zhang, Shiqiang Su, Lizhe Liu, Feifan Song, Xiongjie Cui, Yunpeng Cao, Chao Li, Shen Li, Hanxing He, Yuanhui Kang, Jin Zhang

**Affiliations:** 1 Department of Urology, The People’s Hospital of Shijiazhuang, Shijiazhuang, Hebei, China; 2 Graduate School, Hebei Medical University, Shijiazhuang, Hebei, China; 3 Institute of Medicine and Health, Hebei Medical University, Shijiazhuang, China

**Keywords:** fibrinogen-to-lymphocyte ratio, linear, non-muscle-invasive bladder cancer, predictive indicator, prognosis

## Abstract

**Objective:**

Although the fibrinogen-to-lymphocyte ratio (FLR) is an established prognostic biomarker in various solid tumors, its role in non-muscle-invasive bladder cancer (NMIBC) remains poorly defined. This study aimed not only to investigate the predictive value of preoperative FLR for overall survival (OS) in NMIBC patients undergoing transurethral resection of bladder tumor (TURBt), but also to develop and validate a novel FLR-based nomogram as a practical clinical tool.

**Methods:**

This retrospective study enrolled 304 NMIBC patients who underwent TURBt at the Shijiazhuang People’s Hospital between November 2013 and January 2024, with OS as the primary endpoint. The optimal prognostic cutoff for FLR was determined by maximizing the Youden index via receiver operating characteristic (ROC) curve analysis. Propensity score matching (1:2) was employed to balance baseline confounders. The dose-response relationship between continuous FLR and mortality risk was evaluated using restricted cubic splines (RCS), which confirmed a linear association. Subsequently, independent prognostic factors identified through Cox proportional hazards regression were integrated to construct a nomogram. The model’s predictive accuracy and clinical utility were then comprehensively evaluated using the concordance index (C-index), calibration curves, time-dependent ROC curves, and decision curve analysis (DCA).

**Results:**

The optimal FLR cutoff was identified as 2.91. Patients in the high-FLR group (FLR ≥ 2.91) exhibited significantly poorer OS (P < 0.001) and cancer-specific survival (CSS; P = 0.004). RCS analysis confirmed a significant positive linear association between increasing FLR levels and all-cause mortality risk. Critically, multivariate Cox regression validated FLR as an independent predictor for both OS (Hazard Ratio (HR): 1.520, 95% Confidence Interval (CI): 1.149-2.010) and CSS (HR: 1.536, 95% CI: 1.033-2.284). Integrating FLR into a baseline model improved the C-index for OS prediction from 0.739 to 0.772. The resulting nomogram demonstrated robust discrimination (C-index: 0.772), excellent calibration, and superior net clinical benefit in DCA.

**Conclusion:**

Preoperative FLR is an independent predictor of overall survival in NMIBC, characterized by a robust linear dose-response relationship with mortality risk. This cost-effective biomarker, integrated into our validated nomogram, enhances risk stratification to guide personalized postoperative management.

## 1 Introduction

Bladder cancer represents one of the most prevalent malignancies of the urinary system, ranking as the ninth most common cancer worldwide (1, 2). Non-muscle-invasive bladder cancer (NMIBC) constitutes approximately 75% of cases and is clinically characterized by a high risk of recurrence and potential for progression (3). Although transurethral resection of bladder tumor (TURBt) is the standard treatment, a substantial proportion of patients experience adverse outcomes, leading to highly variable prognoses (4). This clinical heterogeneity underscores the urgent need for robust biomarkers to refine risk stratification and guide individualized postoperative management.

A promising avenue for biomarker discovery lies in the systemic inflammatory and coagulation responses, which play pivotal roles in tumorigenesis (5). The inflammatory microenvironment can foster tumor growth, while the coagulation system may facilitate metastasis (6). Fibrinogen, a key acute-phase reactant, reflects both processes, as it promotes cell adhesion and shields tumor cells from immune attack (7). Conversely, lymphocytes are the core effectors of anti-tumor immunity, and lymphopenia often signifies a compromised host defense (8).

Consequently, the fibrinogen-to-lymphocyte ratio (FLR), which integrates pro-tumoral inflammation (fibrinogen) with anti-tumoral immunity (lymphocytes), is hypothesized to offer a more comprehensive reflection of the host-tumor interplay than either marker alone. While FLR has shown prognostic value in other solid tumors (9–11), its role in NMIBC, particularly its dose-response relationship with survival and its utility within a clinical prediction model, remains to be thoroughly elucidated.

Therefore, this study was designed to evaluate preoperative FLR as an independent predictor of OS in NMIBC patients post-TURBt and, critically, to develop and validate an FLR-based nomogram to translate this biomarker into a clinically applicable tool. The association between FLR and CSS was also explored as a secondary objective.

## 2 Materials and methods

### 2.1 Study population

This retrospective cohort study consecutively enrolled patients with a primary pathological diagnosis of NMIBC who underwent TURBt at the Shijiazhuang People’s Hospital between November 2013 and January 2024. The inclusion criteria were as follows (1): pathologically confirmed primary NMIBC (2); underwent complete TURBt; and (3) had complete clinical, pathological, and follow-up data. Patients were excluded if they met any of the following criteria (1): presence of distant metastases or secondary bladder tumors (2); preoperative active infections or severe liver diseases (e.g., viral hepatitis, cirrhosis) (3); concurrent autoimmune or hematological disorders (4); non-urothelial carcinoma histology (5); missing key preoperative laboratory data (fibrinogen, lymphocyte count) or clinicopathological information (6); incomplete follow-up data or lost to follow-up (7); concurrent active malignancies (8); occurrence of severe perioperative complications; or (9) receipt of regular intravesical instillation chemotherapy with pirarubicin postoperatively. Following these criteria, a total of 304 patients were ultimately included in the study. Among them, 26 patients died, 12 died from bladder cancer, and 278 remained event-free. The study protocol was approved by the Ethics Committee of the Shijiazhuang People’s Hospital and was conducted in accordance with the principles of the Declaration of Helsinki.

Clinical baseline data, including age, sex, smoking history, and comorbidities, were collected from the electronic medical record system. All pathological slides were independently reviewed by two senior pathologists. Tumor grade was determined according to the 2004/2016 World Health Organization (WHO) classification (12), and tumor stage was assigned using the 8th edition of the American Joint Committee on Cancer (AJCC) TNM staging system (2017) (13).

Calculation of FLR, NLR, and PLR Venous blood samples were collected from all patients within one week prior to surgery. Plasma fibrinogen concentration (g/L), Absolute neutrophil count (ANC), Peripheral platelet count and peripheral blood absolute lymphocyte count (×10^9/L) were measured by an automated analyzer at the hospital’s central laboratory. The FLR was calculated using the formula: FLR = Fibrinogen (g/L)/Lymphocyte count (×10^9/L). The NLR was calculated using the formula: NLR = Absolute neutrophil count (×10^9/L)/Lymphocyte count (×10^9/L). The PLR was calculated using the formula: PLR = Peripheral platelet count (×10^9/L)/Lymphocyte count (×10^9/L).

### 2.2 Follow-up and outcomes

All patients underwent postoperative follow-up according to a standard protocol: every 3 months for the first 2 years, every 6 months during years 3 to 5, and annually thereafter. The final follow-up date was March 1, 2025. Follow-up evaluations included routine urinalysis, urinary exfoliative cytology, urinary system ultrasonography, and cystoscopy. The primary endpoint was overall survival (OS), defined as the time from the date of TURBt to death from any cause or the last follow-up. The secondary endpoint was cancer-specific survival (CSS), defined as the time from the date of TURBt to the date of death from bladder cancer or the last follow-up.

### 2.3 Statistical analysis

All statistical analyses were performed using R software (version 4.4.3) and EmpowerStats (version 4.2). A two-sided P-value < 0.05 was considered statistically significant. Continuous variables are presented as median (interquartile range, IQR) and compared using the Mann–Whitney U test, while categorical variables are expressed as frequency (percentage) and analyzed with the Chi-squared test or Fisher’s exact test, as appropriate.

First, to evaluate the predictive performance of FLR, we determined its optimal cutoff for overall survival (OS) using receiver operating characteristic (ROC) curve analysis based on the maximum Youden index. Patients were then stratified into high- and low-FLR groups. To minimize selection bias and balance baseline characteristics, propensity score matching (PSM) was performed using the “MatchIt” package in R. Propensity scores were calculated using a logistic regression model incorporating age, gender, smoking history, diabetes, hypertension, history of abdominal surgery, tumor number, tumor size, tumor grade, and tumor stage. A 1:2 nearest-neighbor matching algorithm was applied with a strict caliper width of 0.02. Covariate balance was assessed using the standardized mean difference (SMD), with an SMD < 0.2 considered indicative of optimal balance.

Survival curves were plotted using the Kaplan-Meier method and compared using the log-rank test. These analyses were conducted in both the entire cohort and the matched cohort to validate the robustness of the findings.

To identify prognostic factors, univariate Cox proportional hazards regression was performed. Variables with P < 0.10 in the univariate analysis were entered into the multivariate Cox regression model to identify independent predictors. Hazard ratios (HRs) and 95% confidence intervals (CIs) were calculated. Similar to the Kaplan-Meier analysis, multivariable Cox regression models were also constructed for the matched cohort to confirm the independent prognostic value of FLR after adjusting for confounders. Restricted cubic splines (RCS) were integrated into multivariable Cox proportional hazards models to explore potential non-linear dose-response relationships between FLR and mortality risk, with tumor grade and tumor stage adjusted as covariates. The Akaike Information Criterion (AIC) was compared across RCS models with 3, 4, and 5 knots; the model yielding the lowest AIC was selected as the optimal fit to test for non-linearity. Additionally, to rigorously verify the linear assumption and test for potential saturation or threshold effects, we performed a threshold effect analysis using a two-piecewise linear regression model. We compared the goodness-of-fit between the standard linear model (one-line model) and the two-piecewise model using the log-likelihood ratio test. A P-value < 0.05 would indicate the presence of a significant inflection point; otherwise, the linear relationship was considered robust. Given the non-significant result of the non-linearity test, which supported a linear assumption, FLR was incorporated into the final Cox model as a continuous linear variable to calculate the hazard ratio (HR) and 95% confidence interval (CI) associated with each unit increase.

Furthermore, based on the optimal cutoff value of FLR, survival curves were plotted using the Kaplan-Meier method for visual validation. Based on the independent predictors identified in the multivariate analysis (P < 0.05), a nomogram was constructed to predict 1- and 3-year OS. The model construction focused on OS, as it was the primary endpoint with a sufficient number of events. A predictive model for CSS was not developed due to the limited number of CSS events, a measure taken to ensure model stability and prevent overfitting.

The nomogram’s discrimination was assessed using the concordance index (C-index) and time-dependent ROC curves. Its calibration and stability were evaluated using calibration plots with 1000-fold bootstrap resampling for internal validation. Finally, decision curve analysis (DCA) was employed to evaluate the clinical net benefit and utility of the nomogram across a range of threshold probabilities.

## 3 Results

### 3.1 Baseline characteristics and propensity score matching

A total of 304 patients were enrolled (**Table 1**; **Supplementary Table 1**), among whom 26 died from all causes and 12 died specifically from bladder cancer. The cohort comprised 268 males and 36 females, with a median age of 66.3 years. The optimal cutoff value for FLR was determined to be 2.91 (Youden’s index = 0.263). Accordingly, patients were stratified into a low-FLR group (n=272, 89.5%) and a high-FLR group (n=32, 10.5%). The median OS was significantly longer in the low-FLR group (71.2 months) compared to the high-FLR group (58.9 months). No significant differences were observed between the two groups in terms of sex, comorbidities, tumor number, tumor size, tumor grade, or T stage (all P > 0.05). A total of 304 patients were enrolled. The optimal cutoff value for FLR was determined to be 2.91. Patients were stratified into a low-FLR group (n=272, 89.5%) and a high-FLR group (n=32, 10.5%). Before matching, significant imbalances were observed, particularly for age (P = 0.007, SMD = 0.419) and smoking history (P = 0.005, SMD = 0.614), with the high-FLR group being older and having a lower prevalence of smoking (**Table 1**).

**Table 1:** Baseline characteristics and propensity score matching.

Variable	Before matching				After matching			
	Low FLR (n=272)	High FLR (n=32)	p value	SMD	Low FLR (n=42)	High FLR (n=24)	p value	SMD
Age, years	67.00 (59.00, 75.00)	73.50 (67.25, 79.25)	0.007	0.419	69.50 (60.25, 77.00)	70.00 (62.25, 77.25)	0.615	0.086
Gender, male (%)	242 (89.0)	26 (81.2)	0.242	0.218	37 (88.1)	21 (87.5)	0.524	0.018
Smoking, yes (%)	91 (33.5)	3 (9.4)	0.005	0.614	7 (16.7)	3 (12.5)	1	0.118
Diabetes, yes (%)	37 (13.6)	3 (9.4)	0.781	0.133	5 (11.9)	3 (12.5)	0.688	0.018
History of abdominal surgery, yes (%)	33 (12.1)	5 (15.6)	0.572	0.101	7 (16.7)	4 (16.7)	1	<0.001
Hypertension, yes (%)	115 (42.3)	17 (53.1)	0.242	0.218	15 (35.7)	10 (41.7)	0.788	0.122
Tumor number, multiple (%)	92 (33.8)	10 (31.2)	0.771	0.055	14 (33.3)	8 (33.3)	0.477	<0.001
Tumor size, >3cm (%)	45 (16.5)	8 (25.0)	0.233	0.210	6 (14.3)	7 (29.2)	0.629	0.367
Tumor grade, high (%)	112 (41.2)	15 (46.9)	0.536	0.115	21 (50.0)	12 (50.0)	0.690	<0.001
Tumor stage, pT1N0M0 (%)	113 (41.5)	16 (50.0)	0.360	0.170	22 (52.4)	11 (45.8)	0.675	0.131

After performing 1:2 PSM with a caliper of 0.02, 24 high-FLR patients were successfully matched with 42 low-FLR patients, resulting in a matched cohort of 66 patients. Post-matching analysis demonstrated significantly improved balance: the SMD for age dropped to 0.086, and for smoking to 0.118. Most covariates achieved an SMD < 0.2, indicating that the potential confounding effects of baseline variables were effectively minimized (**Table 1**).

**Figure 1:**
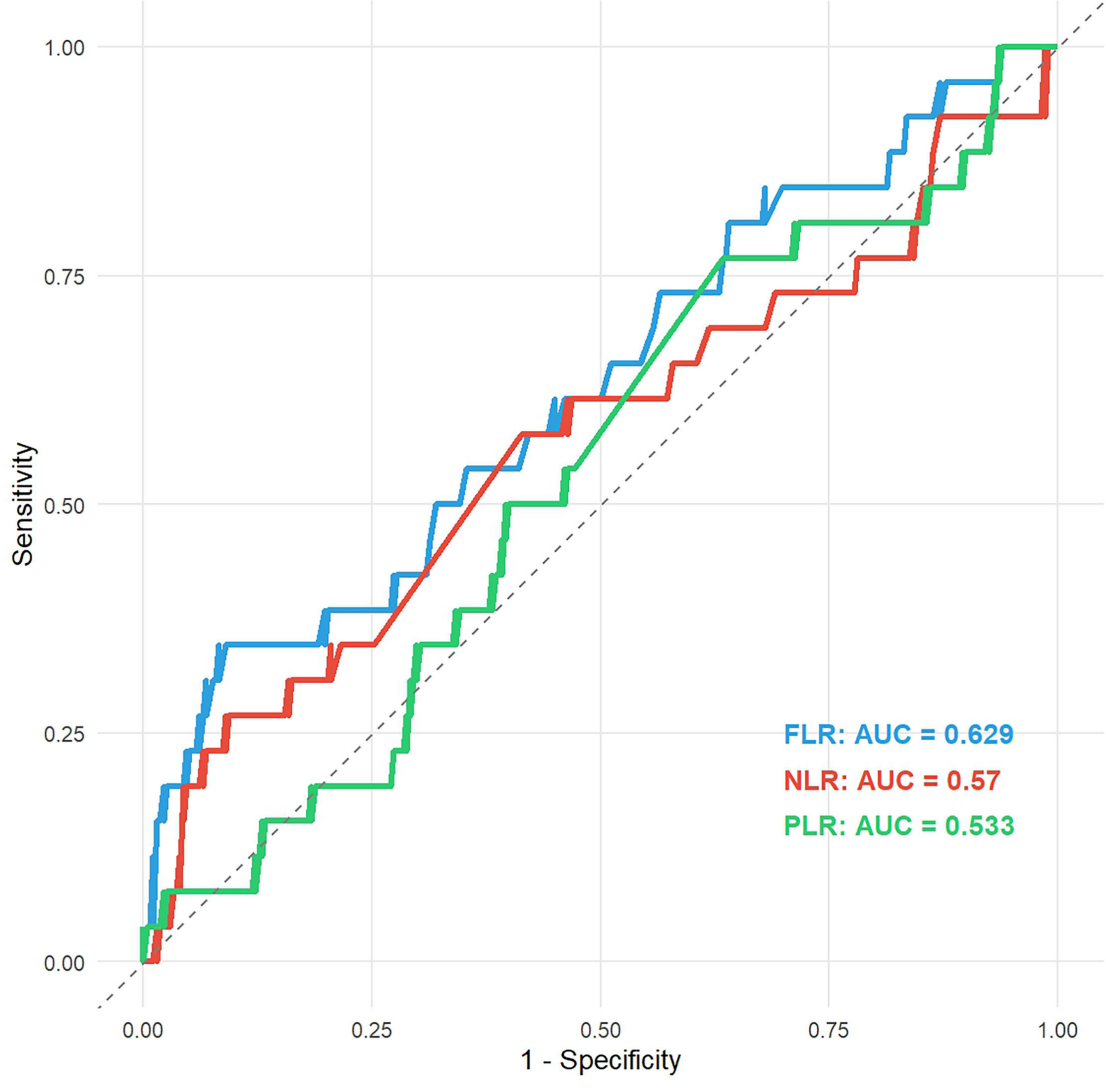
Comparison of ROC curves for FLR, NLR, and PLR. ROC, receiver operating characteristic; FLR, fibrinogen to lymphocyte count Ratio; NLR, absolute neutrophil to lymphocyte count ratio; PLR, peripheral platelet to lymphocyte count ratio.

ROC curve analysis (**Figure 1**) determined the optimal FLR cutoff to be 2.91. At this threshold, FLR (AUC = 0.629) demonstrated superior discriminative ability compared to NLR (AUC = 0.570) and PLR (AUC = 0.533).

### 3.2 Sensitivity analysis in the matched cohort

To validate the robustness of our findings, survival analyses were repeated in the matched cohort. Kaplan-Meier curves reaffirmed that patients in the high-FLR group had significantly worse Overall Survival (OS) compared to the low-FLR group (**Supplementary Figure 1**, P = 0.0037). Multivariate Cox regression in the matched cohort confirmed FLR as a significant independent predictor for OS (HR: 1.53, 95% CI: 1.10–2.14, P = 0.013, **Supplementary Table 2**), independent of tumor grade and stage.

Regarding Cancer-Specific Survival (CSS), while the high-FLR group showed a trend towards poorer survival (**Supplementary Figure 2**), the difference did not reach statistical significance (Log-rank P = 0.078). Similarly, in the multivariate Cox model for the matched cohort, FLR showed an elevated risk (HR: 1.48, 95% CI: 0.94–2.31), but this association was not statistically significant (P = 0.087, **Supplementary Table 3**).

### 3.3 Association of FLR with OS and CSS

**Figure 2:**
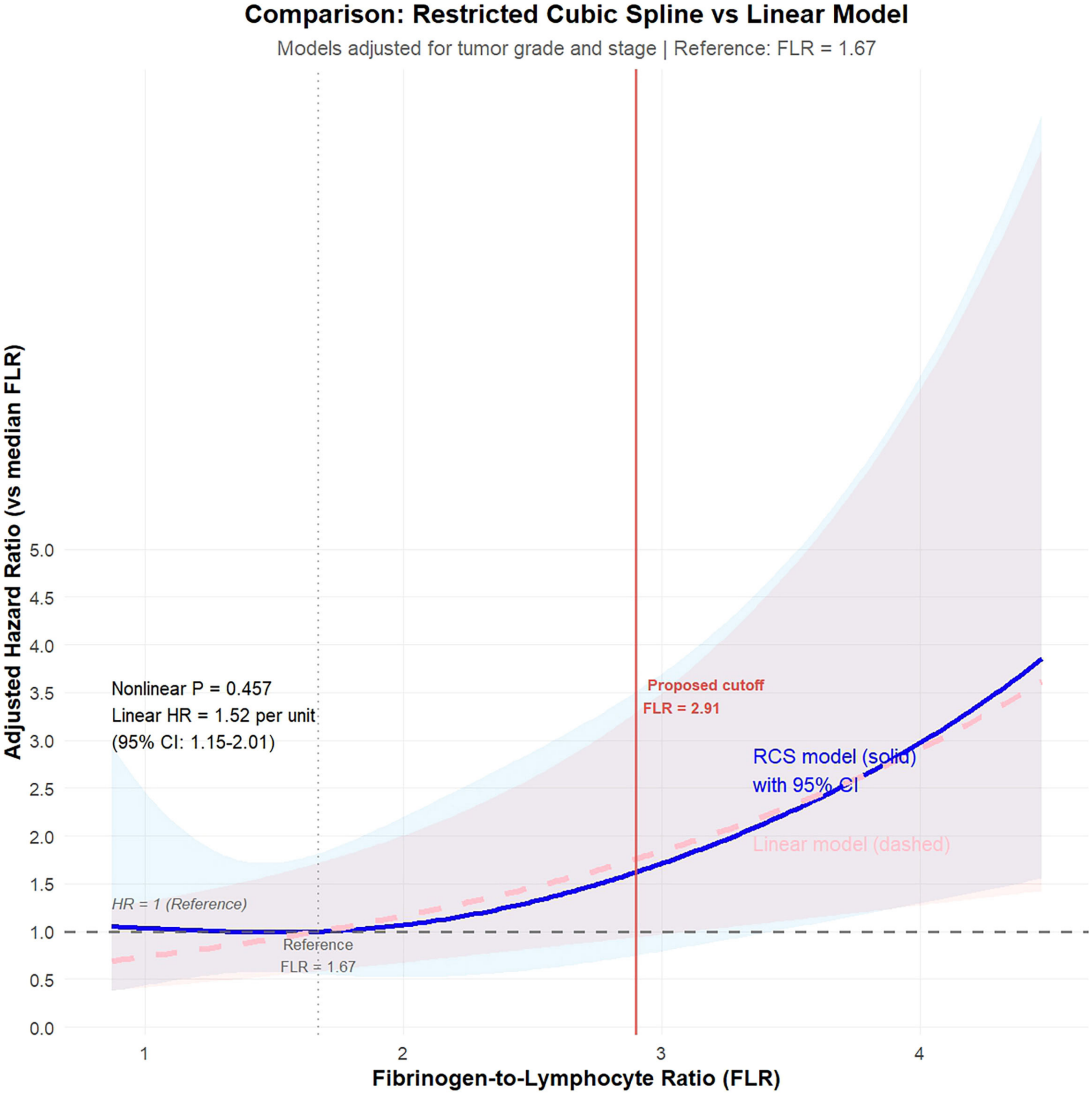
Restricted cubic spline analysis demonstrating linear association between FLR and mortality risk. HR, hazard ratio; CI, confidence interval; RCS, restricted cubic splines.

In the multivariable Cox proportional hazards model adjusted for tumor grade and stage, FLR demonstrated a significant independent association with overall survival in NMIBC patients (Wald χ² = 11.90, p = 0.003). To verify the shape of this relationship, we first fitted a restricted cubic spline (RCS) model with 3 knots, which yielded the lowest AIC value (AIC = 244.39). The results (**Figure 2**) showed no statistically significant non-linear trend between FLR and mortality risk (P for non-linearity = 0.457), indicating an approximate linear relationship. Consequently, treating FLR as a continuous linear variable in the final model, we found that for every 1-unit increase in FLR, the risk of death increased by 52% (HR = 1.52, 95% CI: 1.149 - 2.010, P = 0.003). Consistent with the optimal cutoff derived from the ROC analysis (Youden index), we applied the threshold of 2.91 for risk stratification.

Furthermore, threshold effect analysis corroborated the RCS findings. The log-likelihood ratio test demonstrated that the two-piecewise linear regression model did not significantly improve the model fit compared to the single linear model (P = 0.195). This result confirms that the association between FLR and overall survival is linear across the observed range, with no evidence of a risk threshold or saturation point.

**Figure 3:**
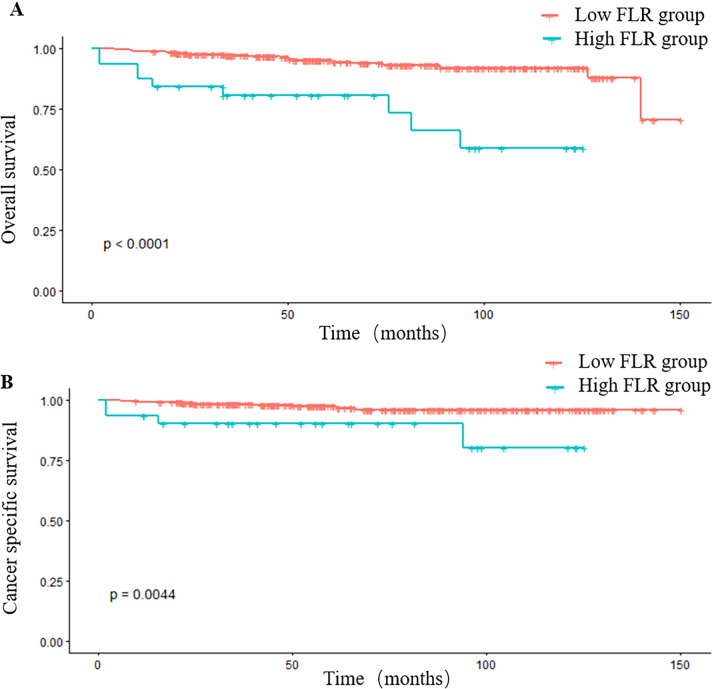
Kaplan–Meier curves for OS **(A)** and CSS **(B)** stratified by the FLR. **(A, B)**, Survival curves for OS **(A)** and CSS **(B)** in all included patients. OS, overall survival; CSS, cancer specific survival; FLR, fibrinogen to lymphocyte count Ratio.

Survival analysis confirmed (**Figure 3A**) that patients with FLR ≥ 2.91 had a significantly shorter median survival (58.9 months) compared to those with FLR < 2.91 (71.2 months), with the difference being highly statistically significant (log-rank P < 0.001).

Kaplan-Meier survival analysis revealed that patients with a high preoperative FLR had a significantly shorter CSS (Log-rank P = 0.0044, **Figure 3B**).

Consequently, FLR was treated as a continuous linear variable in the final multivariate Cox regression models. The analysis confirmed that after adjusting for tumor grade and T stage, FLR remained an independent risk factor for both OS (HR = 1.520, 95% CI: 1.149-2.010, P = 0.003) and CSS (HR = 1.536, 95% CI: 1.033-2.284, P = 0.034). Tumor grade and T stage were also identified as independent prognostic factors for OS (**Tables 2**, **3**).

**Table 2:** Univariate and multivariate Cox regression analysis for overall survival.

Characteristic	Univariate analysis		Multivariate analysis	
	Hazard ratio (95%)	P value	Hazard ratio (95%)	P value
Gender				
Female	Reference			
Male	0.523 (0.196-1.396)	0.196		
Age				
≤60	Reference			
>60	11.302 (1.531-83.447)	0.017	6.408 (0.841-48.815)	0.073
Diabetes				
No	Reference			
Yes	1.881 (0.704-5.026)	0.208		
History of abdominal surgery				
No	Reference			
Yes	0.743 (0.174-3.165)	0.688		
Hypertension				
No	Reference			
Yes	1.267 (0.573-2.756)	0.568		
Smoking				
No	Reference			
Yes	0.647 (0.260-1.614)	0.351		
Tumor number				
Single	Reference			
Multiple	1.229 (0.557-2.711)	0.609		
Tumor size				
≤3cm	Reference			
>3cm	2.117 (0.881-5.090)	0.094		
Tumor grade				
Low	Reference			
High	4.622 (1.855-11.515)	0.001	2.938 (1.161-7.437)	0.023
Tumor stage				
pTaN0M0	Reference			
pT1N0M0	3.765 (1.502-9.439)	0.005	3. 357 (1.340-8.411)	0.010
FLR	1.701 (1.300-2.226)	<0.001	1.520 (1.149-2.010)	0.003

**Table 3:** Univariate and multivariate Cox regression analysis for cancer-specific survival (CSS).

Characteristic	Univariate analysis		Multivariate analysis	
	Hazard ratio (95%)	P value	Hazard ratio (95%)	P value
Gender				
Female	Reference			
Male	0.273 (0.082-0.906)	0.034	0.218 (0.063-0.756)	0.016
Age				
≤60	Reference			
>60	36.905 (0.223-6106.996)	0.166		
Diabetes				
No	Reference			
Yes	2.502 (0.675-9.270)	0.170		
History of abdominal surgery				
No	Reference			
Yes	0.723 (0.093-5.612)	0.757		
Hypertension				
No	Reference			
Yes	1.358 (0.438-4.211)	0.596		
Smoking				
No	Reference			
Yes	1.078 (0.324-3.580)	0.903		
Tumor number				
Single	Reference			
Multiple	1.409 (0.447-4.441)	0.559		
Tumor size				
≤3cm	Reference			
>3cm	1.729 (0.467-6.394)	0.412		
Tumor grade				
Low	Reference			
High	7.069 (1.549-32.269)	0.012	6.464 (1.404-29.760)	0.017
Tumor stage				
pTaN0M0	Reference			
pT1N0M0	6.172 (1.347-28.292)	0.019	8.013 (1.598-40.173)	0.011
FLR	4.835 (1.454-16.072)	0.010	1.536 (1.033-2.284)	0.034

### 3.4 Development and performance of the nomogram

**Figure 4:**
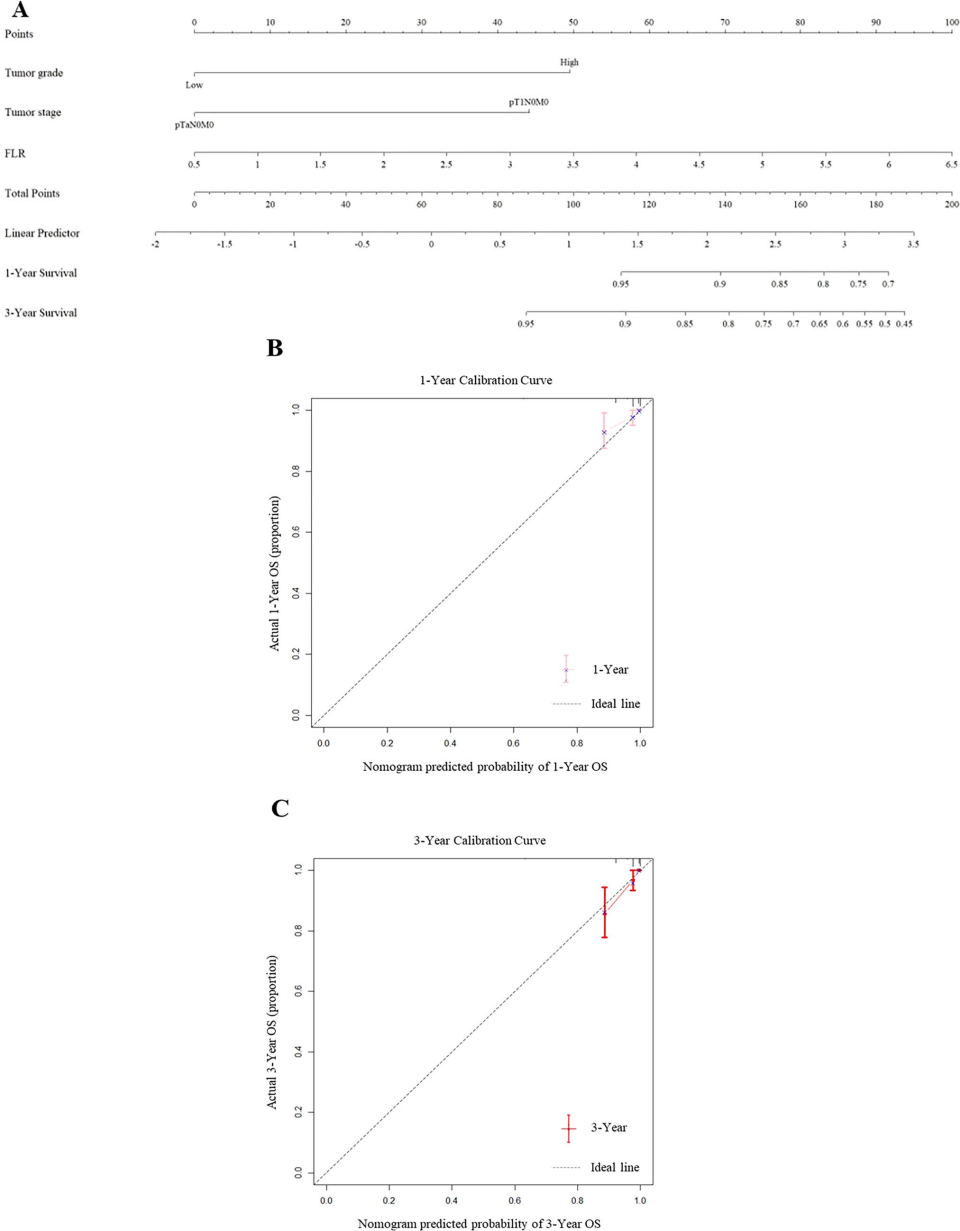
Nomograms and calibration curves for the prediction of 1- and 3-year OS. Nomograms for 1- and 3-year OS **(A)** prediction. **(B, C)** Calibration curves for estimating the prediction of 1-year **(B)** and 3-year **(C)** OS between the prediction and the actual observation. OS, overall survival; FLR, fibrinogen to lymphocyte count Ratio.

Based on the three independent predictors of OS identified from the multivariate analysis (tumor grade, T stage, and FLR), we developed a nomogram to predict 1- and 3-year OS (**Figure 4A**).

The nomogram demonstrated strong predictive performance. Its concordance index (C-index) was 0.772 (**Table 4**), which was significantly higher than that of a baseline model without FLR (C-index = 0.739), indicating that the inclusion of FLR improved the model’s discriminative ability. Internal validation using 1,000 bootstrap resamples yielded a corrected C-index of 0.739, suggesting good model stability.

**Table 4:** C- Index of the nomogram for the prediction of survival outcomes.

Outcome	Models	C-index	Optimism	95%CI	Corrected C-index	ΔC-index
Overall Survival	Nomogram without FLR	0.739	0.025	0.645-0.833	0.727	
	Nomogram*	0.772	0.029	0.684-0.860	0.758	
	Nomogram without FLR vs Nomogram					0.031

*Tumor grade, Tumor stage, FLR group were included in the nomogram for overall survival prediction.

FLR, fibrinogen to lymphocyte count Ratio; CI, confidence interval.

**Figure 5:**
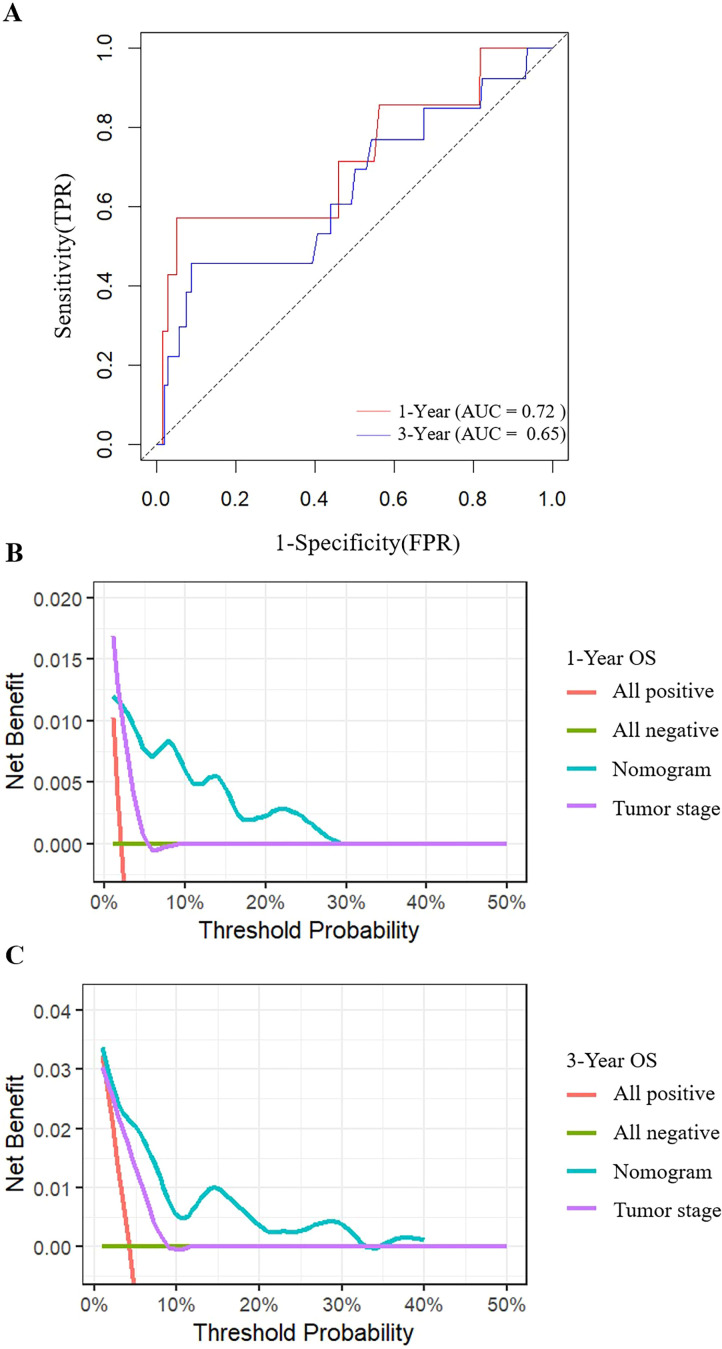
ROC curves and decision curve analyses of the nomogram for OS prediction. ROC curves for OS **(A)**. **(B, C)**, Decision curve analyses for 1-year **(B)** and 3-year **(C)** OS prediction. ROC, receiver operating characteristic; OS, overall survival; FLR, fibrinogen to lymphocyte count Ratio.

The calibration plots for 1-year (**Figure 4B**) and 3-year (**Figure 4C**) OS showed excellent agreement between the nomogram-predicted probabilities and the actual observed outcomes, confirming the model’s high calibration accuracy. Furthermore, time-dependent ROC analysis yielded an AUC of 0.72 for 1-year OS and 0.65 for 3-year OS (**Figure 5A**), verifying its stable predictive efficacy at different time points.

Moreover, decision curve analysis (DCA) demonstrated that using our nomogram for clinical decision-making provided a greater net benefit than either the “treat-all” or “treat-none” strategies across a wide range of threshold probabilities (5% to 40%). The nomogram also outperformed a model based on T stage alone, highlighting its superior clinical utility (**Figures 5B, C**).

## 4 Discussion

In this study, we investigated the prognostic value of preoperative FLR in 304 patients with NMIBC. Our findings align with previous research demonstrating the prognostic utility of FLR in other malignancies, such as esophageal cancer (14), hepatocellular carcinoma (15), and gastric cancer (10). We identified an optimal FLR cutoff of 2.91. Notably, FLR demonstrated a higher AUC compared to traditional markers (16) like NLR and PLR, suggesting that integrating coagulation and immune pathways provides a more comprehensive reflection of the host-tumor interaction.

After grouping, only 32 patients (10.5%) were in the high FLR group compared to 272 in the low FLR group. This extreme imbalance could reduce statistical power, and the low number of events in the 32 cases might lead to unstable multivariate regression results. Given the retrospective nature of this study and the uneven distribution of baseline characteristics—specifically that patients in the high-FLR group were significantly older (P = 0.007)—we employed Propensity Score Matching (PSM) as a rigorous sensitivity analysis. Using a strict caliper (0.02), we successfully balanced key confounders, reducing the Standardized Mean Difference (SMD) for age from 0.419 to 0.086. In this well-balanced matched cohort, FLR remained a statistically significant independent risk factor for Overall Survival (HR = 1.53, P = 0.013). This finding is crucial, as it confirms that the prognostic value of FLR is not merely a proxy for advanced age or other comorbidities but reflects an independent biological risk.

It is noteworthy that while FLR was significantly associated with Cancer-Specific Survival (CSS) in the entire cohort (P = 0.034), this significance was not maintained in the matched cohort (P = 0.087). This discrepancy is likely attributable to the reduced statistical power inherent in the smaller matched sample size (n=66) combined with the low number of cancer-specific death events. However, the Hazard Ratio for CSS in the matched cohort (HR = 1.48) remained comparable to that of the full cohort (HR = 1.54), suggesting a consistent direction of risk. This implies that the association between FLR and cancer-specific mortality likely exists but requires a larger sample size to be statistically confirmed in a matched setting.

We further excluded significant non-linear relationships via Restricted Cubic Spline (RCS) analysis, thereby enhancing the statistical validity of using linear models and a single cut-off value. Our study rigorously explored the dose-response relationship between FLR and prognosis. Both Restricted Cubic Spline (RCS) analysis and threshold effect analysis (P = 0.195) consistently supported a linear model. This implies that the risk of mortality increases continuously and proportionally with rising FLR levels, rather than acting as a binary switch. This justifies the inclusion of FLR as a continuous linear predictor in our nomogram, preserving its full prognostic information. Kaplan-Meier survival analysis clearly demonstrated that Overall Survival (OS) and Cancer-Specific Survival (CSS) were significantly shorter in the high FLR group compared to the low FLR group. The RCS analysis confirmed a robust and significant linear dose-response relationship between continuously increasing FLR values and the risk of all-cause mortality. This finding moves beyond simple binary risk classification, revealing that every incremental increase in FLR corresponds to a progressively higher risk of death, highlighting its value as a dynamic and sensitive biomarker.

In multivariate Cox regression analysis, FLR was identified as an independent prognostic factor for OS (HR: 1.520, 95% CI: 1.149–2.010, P = 0.003) and CSS (HR: 1.536, 95% CI: 1.033–2.284, P = 0.034). This independence from established clinicopathological factors such as tumor grade and stage highlights its unique contribution to prognostic stratification. Notably, tumor grade and stage remained significant independent predictors, reinforcing their importance in NMIBC prognosis. Considering that the number of disease-specific deaths from non-muscle-invasive bladder cancer in this cohort is extremely limited (n=12), the sample size is insufficient to support the construction of a stable and statistically robust multivariable nomogram model. To avoid model overfitting and prediction bias due to sparse data, this study decided not to develop a nomogram for CSS, and only assessed the correlation between FLR and CSS using the Cox regression model. Integrating FLR with these traditional prognostic factors into a nomogram represents a significant advancement. Our nomogram, incorporating tumor grade, stage, and FLR, exhibited superior discriminatory ability with a C-index of 0.772, significantly outperforming the baseline model without FLR (C-index = 0.739). Time-dependent AUC values of 0.72 for 1-year OS and 0.65 for 3-year OS further confirmed the robust predictive performance of the nomogram across different time frames. Internal validation using 1000 bootstrap resamples confirmed excellent calibration for predicting 1-year and 3-year OS, indicating high consistency between predicted and actual survival probabilities. Incorporating FLR as a continuous variable into the prognostic nomogram, combined with established clinicopathological factors, creates a model with superior predictive accuracy and clinical utility. The enhanced discriminatory power, validated by improved C-indices and robust time-dependent AUC values, coupled with compelling evidence of clinical net benefit in Decision Curve Analysis (DCA), underscores the practical value of adding FLR for individualized risk stratification. As a biomarker derived from routine, cost-effective preoperative blood tests, FLR offers a highly accessible and objective tool that can be seamlessly integrated into standard clinical practice.

The first component of FLR is fibrinogen. In recent years, significant progress has been made in understanding the relationship between inflammation and cancer, revealing that inflammation plays a substantial role in tumor development and often pre-exists in the early stages of many malignancies. Additionally, studies indicate that patients with malignancies are often in a hypercoagulable state, which is associated not only with thrombosis but also with tumor progression. Fibrinogen is considered a critical coagulation factor and acute-phase protein, serving as a marker reflecting both inflammation and hypercoagulability, and is significantly elevated in the serum of patients with various malignancies and inflammatory diseases (15, 16). Yang et al. (17), studying 145 patients after radical cystectomy for bladder cancer, found that higher plasma fibrinogen levels were associated with poorer prognosis, and the prognostic ability of plasma fibrinogen was superior to other coagulation and fibrinolytic factors. Multivariate Cox regression showed plasma fibrinogen was an independent predictor for OS (HR = 2.58, 95% CI = 1.28–5.23, p = 0.008) and DFS (HR = 2.60, 95% CI = 1.20–5.65, p = 0.016). Zhang et al. (18), studying 283 patients with NMIBC treated by transurethral resection, found significant correlations between higher fibrinogen values and higher tumor stage (P = 0.030) and histological grade (P = 0.000). The 5-year recurrence-free survival and progression-free survival rates were 63.5% and 83.8%, respectively. Uni- and multivariate analyses identified higher fibrinogen values as independent risk factors affecting tumor recurrence and progression. A meta-analysis by Axel John et al. (19) indicated that genitourinary tumors have procoagulant functions, particularly in prostate, bladder, and renal cancers. This may increase the risk of vascular thrombosis and lead to metastatic progression. Clinical studies have linked elevated hemostatic biomarkers to poor prognosis in genitourinary cancer patients; thus, anticoagulation might have therapeutic effects beyond thrombosis prevention. A prospective study by Aristeidis Alevizopoulos et al. (20) found elevated fibrinogen in bladder cancer was associated with higher tumor stages (II–IV). Fibrinogen plays an important role in the occurrence and development of malignant tumors, potentially through the following mechanisms: malignant tumor cells typically possess high levels of fibrinogen receptors, namely intercellular adhesion molecule-1 (ICAM-1), while platelets also possess integrin fibrinogen receptors. Ultimately, malignant tumor cells bind to platelets via fibrinogen to form aggregates. These aggregates form and bind to the endothelium of other organs, promoting tumor progression and metastasis. These aggregates can also adhere to the surface of malignant tumor cells, wrapping them to protect the tumor cells and helping them evade the human immune system. Through these mechanisms, fibrinogen plays a role in the recurrence, progression, and even death associated with bladder cancer.

The second component of FLR is the lymphocyte count, which plays an extremely important role in the immune response against malignant tumors. Related studies have found that lymphocytes possess powerful anti-tumor immune functions and can inhibit the progression of various tumors; lymphopenia is often regarded as immune deficiency and has an adverse effect on the prognosis of many tumors (21). It has been reported that tumors with high expression of certain lymphocytes, such as CD8+ T lymphocytes, play an important role in the tumor immune process, and these tumors often have a better prognosis. Furthermore, a study (22) pointed out that pretreatment lymphopenia is an independent poor prognostic factor in muscle-invasive and advanced bladder cancer. This may be a manifestation of cancer-induced immunosuppression driving tumor progression. However, the potential mechanisms between lymphopenia and tumor progression or poor prognosis remain complex and require further experimental investigation.

By calculating the Fibrinogen-to-Lymphocyte Ratio (FLR), we combined fibrinogen and lymphocytes in hopes of obtaining a better and more effective prognostic indicator. Li et al. (15) retrospectively analyzed clinical data from 479 patients with hepatocellular carcinoma (HCC) undergoing radical resection and found that FLR was an independent predictor of postoperative OS (p = 0.002) and PFS (p = 0.001). Fan et al. (14) conducted a study on FLR for predicting prognosis in patients with esophageal squamous cell carcinoma (ESCC) after radical esophagectomy, collecting data from 673 ESCC patients; results showed high FLR was an independent predictor of OS (HR: 1.448, 95% CI: 1.073–1.952, P = 0.015) and DFS (HR: 1.445, 95% CI: 1.084–1.925, P = 0.012). By collecting clinical data from 304 patients with non-muscle invasive bladder cancer, this study concluded that FLR is an independent risk factor affecting OS in NMIBC patients, which is consistent with the findings of Fan and Li et al.

Fibrinogen is an important coagulation factor and inflammatory protein; its elevation reflects the body’s hypercoagulable state and inflammatory infiltration, which are conducive to tumor occurrence and development. Lymphocytes are a critical marker primarily reflecting the body’s immune function; their reduction often indicates immune regulation disorders, which are also conducive to tumor occurrence and development. When fibrinogen increases and lymphocytes decrease—that is, when FLR increases—patients often have a poorer prognosis.

Our study has several limitations. First, it is a single-center retrospective study, which inherently carries the risk of selection bias and limits the generalizability of our findings. Larger-scale, multi-center prospective cohort studies would help validate these results externally. Second, although we adjusted for several confounding factors, residual confounding from unmeasured variables cannot be entirely ruled out. For instance, detailed information on unexcluded systemic inflammatory diseases or specific comorbidities affecting inflammatory markers could help refine the analysis. Third, the study population was predominantly male, reflecting the epidemiology of bladder cancer, but this limits representativeness for female patients. Fourth, this study focused on Overall Survival (OS) and Cancer-Specific Survival (CSS), but future research could explore the predictive value of FLR for Recurrence-Free Survival and Progression-Free Survival, which are also critical outcome metrics for NMIBC. Finally, while our nomogram showed good internal validation, external validation in independent cohorts is crucial to confirm its robustness and clinical applicability across different patient populations and medical settings.

In summary, this study provides strong evidence establishing preoperative serum FLR as an independent prognostic biomarker for OS and CSS in patients with Non-Muscle Invasive Bladder Cancer (NMIBC) undergoing transurethral resection of bladder tumor. We observed for the first time that higher FLR is associated with poorer overall survival (OS). The nomogram based on continuous FLR offers a more precise model for individual risk stratification, holding great potential for guiding personalized surveillance and optimizing adjuvant treatment decisions. Further research is required to validate our results.

## 5 Conclusion

Preoperative FLR is an independent predictor of poor survival in NMIBC patients, and its clear linear relationship with mortality risk underscores its value as a robust prognostic tool. Building on this, our FLR-based nomogram provides a precise and practical tool for enhanced risk stratification, holding significant potential to guide personalized surveillance and optimize treatment strategies.

## Data Availability

The raw data supporting the conclusions of this article will be made available by the authors, without undue reservation.
