# Supplementary material for: Preoperative fibrinogen-to-lymphocyte ratio as a prognostic biomarker for non-muscle-invasive bladder cancer
Source: Front Oncol. 2026 Jan 22;16:1707696. doi: 10.3389/fonc.2026.1707696 (PMC12872508; doi:10.3389/fonc.2026.1707696)
Supplement: Supplementary file 1 [file Table1.docx]

Supplementary Material

# Supplementary Figures and Tables

## Supplementary Tables

Supplementary Table 1 Study Characteristics

| **Characteristics** | **Low FLR**  (n = 272) | **High FLR**  (n = 32) | ***P* value** |
| --- | --- | --- | --- |
| Age, year | 65.74 (12.09) | 70.91 (12.54) | 0.007 |
| FLR | 1.65 (0.50) | 3.93 (0.99) | <0.001 |
| NLR | 2.61 (1.73) | 3.27 (1.95) | 0.008 |
| PLR | 128.80 (46.31) | 163.87 (60.86) | 0.002 |
| Gender, n (%) |  |  | 0.242 |
| Female | 30 (11%) | 6 (19%) |  |
| Male | 242 (89%) | 26 (81%) |  |
| History of abdominal surgery, n (%) |  |  | 0.572 |
| No | 239 (88%) | 27 (84%) |  |
| Yes | 33 (12%) | 5 (16%) |  |
| Hypertension, n (%) |  |  | 0.242 |
| No | 157 (58%) | 15 (47%) |  |
| Yes | 115 (42%) | 17 (53%) |  |
| Diabetes, n (%) |  |  | 0.781 |
| No | 235 (86%) | 29 (91%) |  |
| Yes | 37 (14%) | 3 (9.4%) |  |
| Smoking, n (%) |  |  | 0.005 |
| No | 181 (67%) | 29 (91%) |  |
| Yes | 91 (33%) | 3 (9.4%) |  |
| Tumor number, n (%) |  |  | 0.771 |
| Single | 180 (66%) | 22 (69%) |  |
| Multiple | 92 (34%) | 10 (31%) |  |
| Tumor size, n (%) |  |  | 0.233 |
| ≤3cm | 227 (83%) | 24 (75%) |  |
| ＞3cm | 45 (17%) | 8 (25%) |  |
| Tumor grade, n (%) |  |  | 0.536 |
| Low grade | 160 (59%) | 17 (53%) |  |
| High grade | 112 (41%) | 15 (47%) |  |
| Tumor stage, n (%) |  |  | 0.36 |
| pTaN0M0 | 159 (58%) | 16 (50%) |  |
| pT1N0M0 | 113 (42%) | 16 (50%) |  |
