# Supplementary material for: Preoperative fibrinogen-to-lymphocyte ratio as a prognostic biomarker for non-muscle-invasive bladder cancer
Source: Front Oncol. 2026 Jan 22;16:1707696. doi: 10.3389/fonc.2026.1707696 (PMC12872508; doi:10.3389/fonc.2026.1707696)
Supplement: Supplementary file 2 [file Table2.docx]

Supplementary Material

# Supplementary Figures and Tables

## Supplementary Tables

Supplementary Table 2 Univariate and Multivariate Cox Regression Analysis for Overall Survival (After Matched)

| Characteristic | Univariate Analysis | | Multivariate Analysis | |
| --- | --- | --- | --- | --- |
|  | Hazard Ratio(95%) | P value | Hazard Ratio(95%) | P value |
| Gender |  |  |  |  |
| Female | Reference |  |  |  |
| Male | 0.58(0.15- 2.21) | 0.429 |  |  |
| Age |  |  |  |  |
| ≤60 | Reference |  |  |  |
| ＞60 | 30.84(0.08-11856.634) | 0.259 |  |  |
| Diabetes |  |  |  |  |
| No | Reference |  |  |  |
| Yes | 2.87(0.61- 13.5) | 0.182 |  |  |
| History of abdominal surgery |  |  |  |  |
| No | Reference |  |  |  |
| Yes | 0.57(0.07- 4.43) | 0.587 |  |  |
| Hypertension |  |  |  |  |
| No | Reference |  |  |  |
| Yes | 2.48(0.72- 8.52) | 0.15 |  |  |
| Smoking |  |  |  |  |
| No | Reference |  |  |  |
| Yes | 0.63(0.08- 4.96) | 0.662 |  |  |
| Tumor number |  |  |  |  |
| Single | Reference |  |  |  |
| Multiple | 1.14(0.35- 3.74) | 0.828 |  |  |
| Tumor size |  |  |  |  |
| ≤3cm | Reference |  |  |  |
| ＞3cm | 0.98(0.21- 4.56) | 0.982 |  |  |
| Tumor grade |  |  |  |  |
| Low | Reference |  |  |  |
| High | 3.76(0.99- 14.3) | 0.052 | 2.92(0.73- 11.7) | 0.13 |
| Tumor stage |  |  |  |  |
| pTaN0M0 | Reference |  |  |  |
| pT1N0M0 | 2.28(0.60- 8.69) | 0.226 |  |  |
| FLR | 1.69(1.20- 2.38) | 0.003 | 1.53(1.10- 2.14) | 0.013 |
