# Supplementary material for: Preoperative fibrinogen-to-lymphocyte ratio as a prognostic biomarker for non-muscle-invasive bladder cancer
Source: Front Oncol. 2026 Jan 22;16:1707696. doi: 10.3389/fonc.2026.1707696 (PMC12872508; doi:10.3389/fonc.2026.1707696)
Supplement: Supplementary file 3 [file Table3.docx]

Supplementary Material

# Supplementary Figures and Tables

## Supplementary Tables

Supplementary Table 3 Univariate and Multivariate Cox Regression Analysis for Cancer-Specific survival (After Matched)

| Characteristic | Univariate Analysis | | Multivariate Analysis | |
| --- | --- | --- | --- | --- |
|  | Hazard Ratio(95%) | P value | Hazard Ratio(95%) | P value |
| Gender |  |  |  |  |
| Female | Reference |  |  |  |
| Male | 0.41(0.07-2.23) | 0.302 |  |  |
| Age |  |  |  |  |
| ≤60 | Reference |  |  |  |
| ＞60 | 30.61(0.01-103005.325) | 0.409 |  |  |
| Diabetes |  |  |  |  |
| No | Reference |  |  |  |
| Yes | 5.58(1.01- 30.9) | 0.049 | 3.15(0.51- 19.6) | 0.218 |
| History of abdominal surgery |  |  |  |  |
| No | Reference |  |  |  |
| Yes | 1.06(0.12- 9.05) | 0.96 |  |  |
| Hypertension |  |  |  |  |
| No | Reference |  |  |  |
| Yes | 1.32(0.27- 6.59) | 0.733 |  |  |
| Smoking |  |  |  |  |
| No | Reference |  |  |  |
| Yes | 1.27(0.15- 11.0) | 0.827 |  |  |
| Tumor number |  |  |  |  |
| Single | Reference |  |  |  |
| Multiple | 1.38(0.28- 6.86) | 0.691 |  |  |
| Tumor size |  |  |  |  |
| ≤3cm | Reference |  |  |  |
| ＞3cm | 0.83(0.10- 7.12) | 0.866 |  |  |
| Tumor grade |  |  |  |  |
| Low | Reference |  |  |  |
| High | 6.67(0.77- 57.9) | 0.085 | 4.36(0.45- 42.5) | 0.205 |
| Tumor stage |  |  |  |  |
| pTaN0M0 | Reference |  |  |  |
| pT1N0M0 | 4.57(0.53- 39.3) | 0.167 |  |  |
| FLR | 1.73(1.08- 2.77) | 0.022 | 1.48(0.94- 2.31) | 0.087 |
