# Supplementary material for: Preoperative fibrinogen-to-lymphocyte ratio as a prognostic biomarker for non-muscle-invasive bladder cancer
Source: Front Oncol. 2026 Jan 22;16:1707696. doi: 10.3389/fonc.2026.1707696 (PMC12872508; doi:10.3389/fonc.2026.1707696)
Supplement: Supplementary file 4 [file SupplementaryFile1.docx]

Supplementary Material

# Supplementary Figures and Tables

## Supplementary Figure


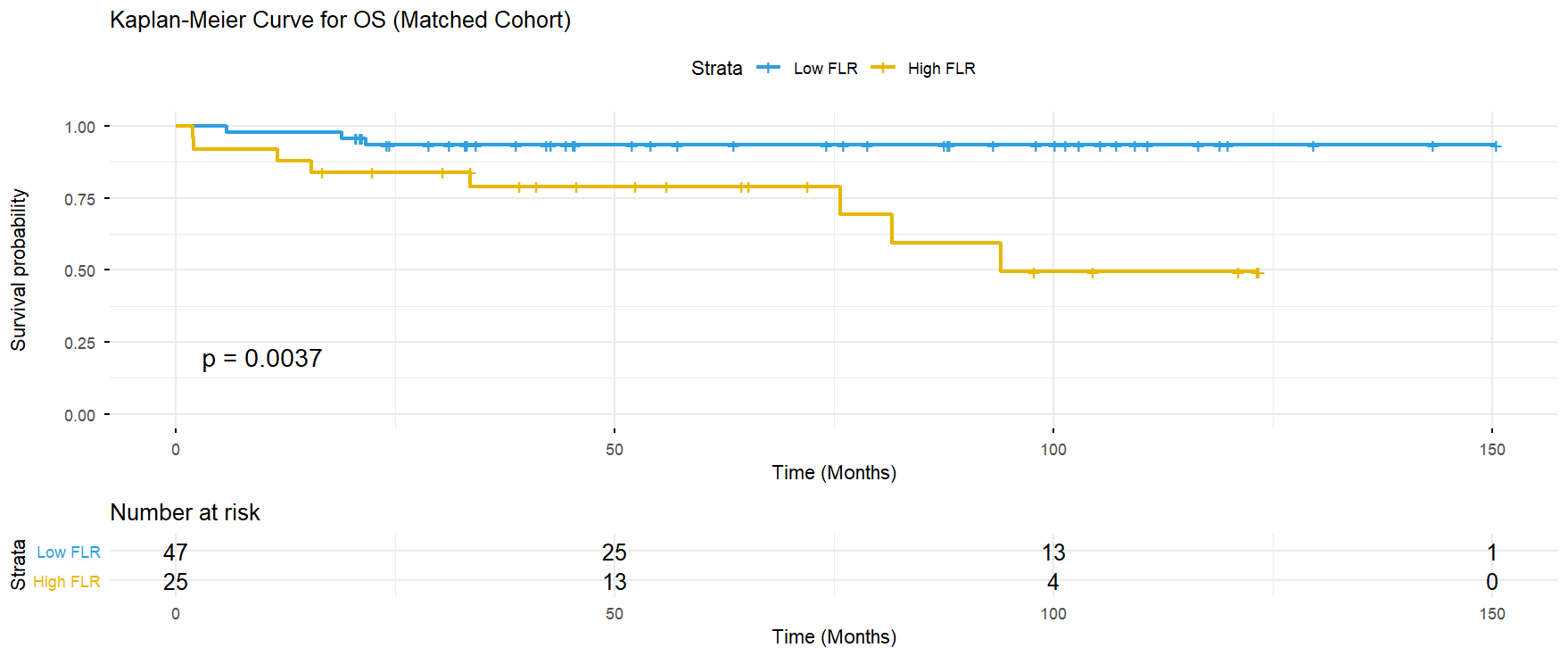


Supplementary Figure 1 Kaplan–Meier curves for OS (A) and CSS (B) stratified by the FLR(Matched Cohort).

Abbreviations: OS, overall survival; FLR, fibrinogen to lymphocyte count Ratio.
