# Supplementary material for: Preoperative fibrinogen-to-lymphocyte ratio as a prognostic biomarker for non-muscle-invasive bladder cancer
Source: Front Oncol. 2026 Jan 22;16:1707696. doi: 10.3389/fonc.2026.1707696 (PMC12872508; doi:10.3389/fonc.2026.1707696)
Supplement: Supplementary file 5 [file SupplementaryFile2.docx]

Supplementary Material

# Supplementary Figures and Tables

## Supplementary Figure


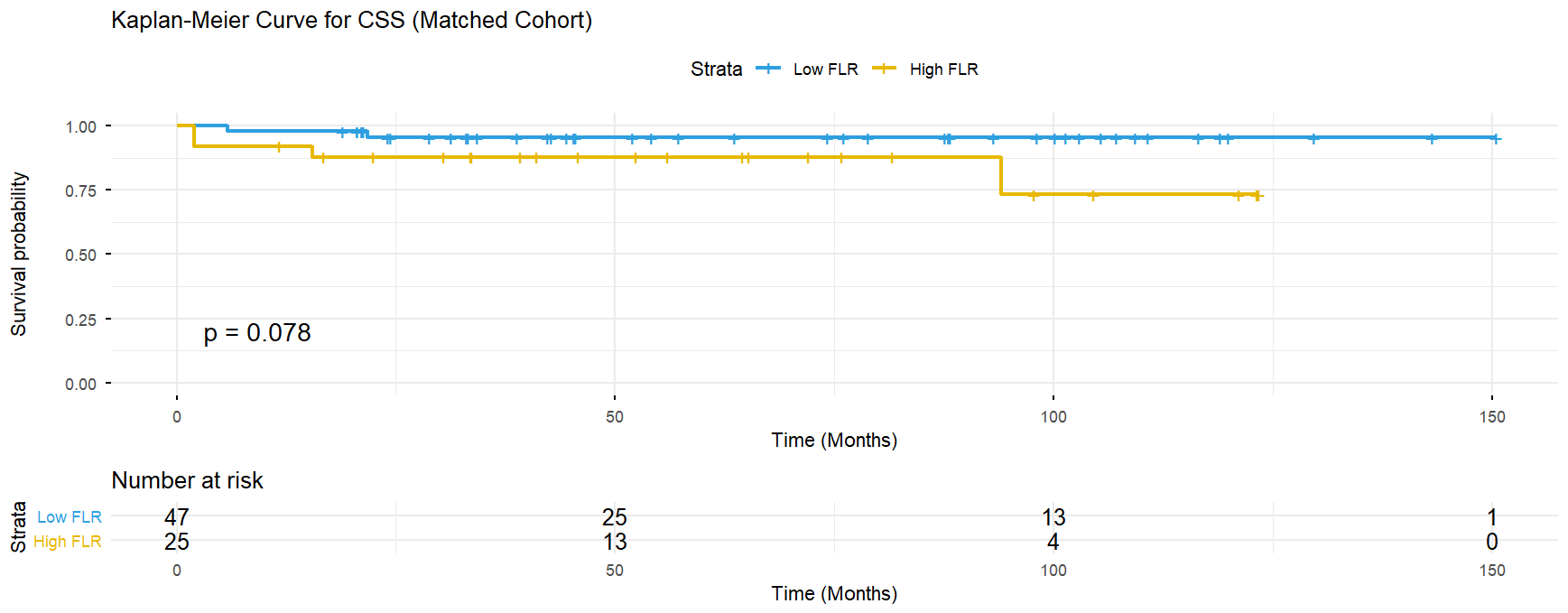


Supplementary Figure 2 Kaplan–Meier curves for CSS stratified by the FLR (Matched Cohort).

Abbreviations: CSS, cancer specific survival; FLR, fibrinogen to lymphocyte count Ratio.
